# Supplementary figures and images for: Dichloroacetate-induced metabolic reprogramming improves lifespan in a Drosophila model of surviving sepsis
Source: PLoS One. 2020 Nov 5;15(11):e0241122. doi: 10.1371/journal.pone.0241122 (PMC7643993; doi:10.1371/journal.pone.0241122)

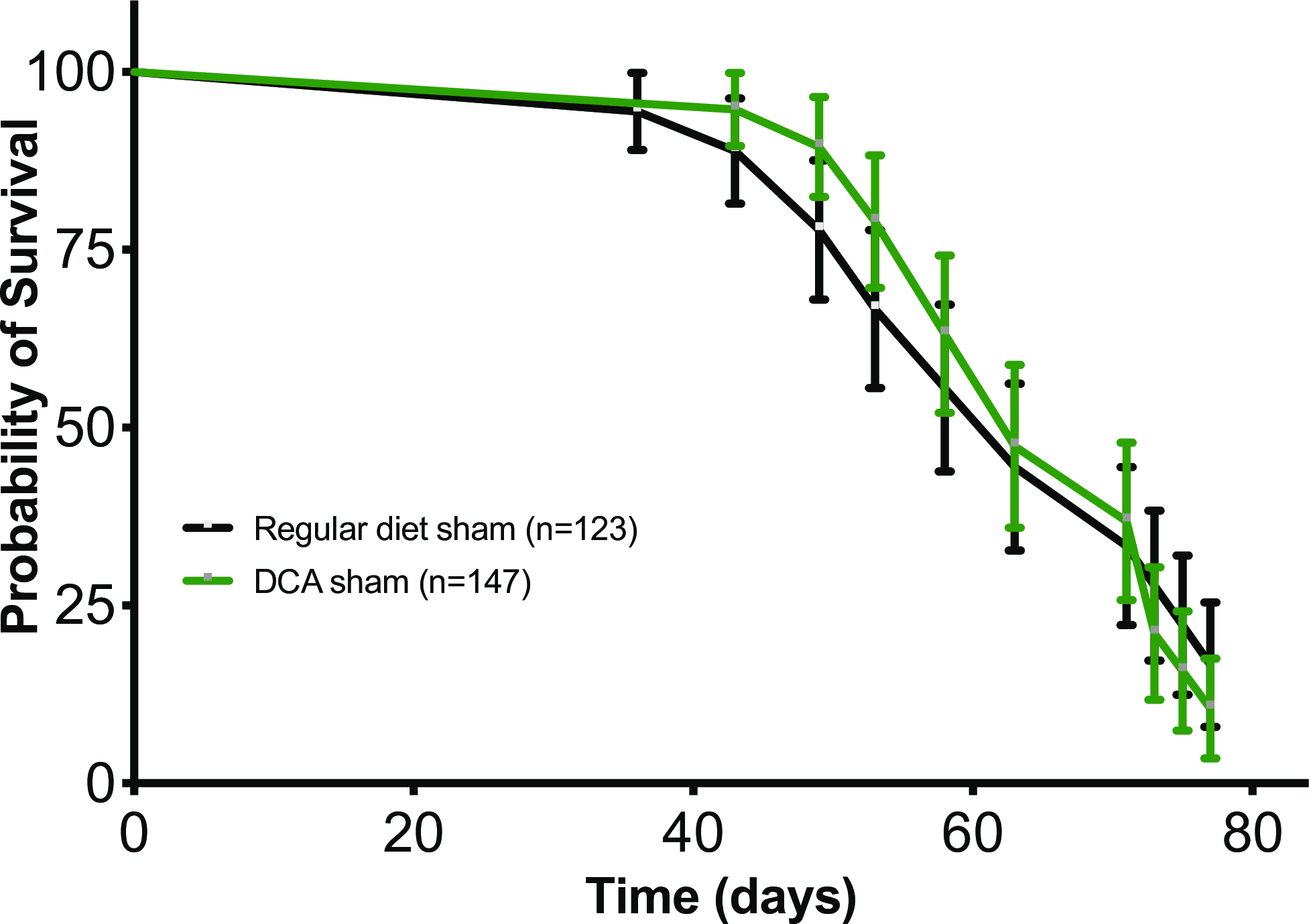

Supplement: S1 Fig — To study the impact of DCA in diet, sterile needle injured (“sham”) flies were divided to either receive regular or DCA diet. Survival of Drosophila melanogaster after sterile injury was assessed following the initial 4–6 hours to exclude trauma-associated mortality. All flies received oral linezolid (0.5 mg/mL) for 18 h. Lifespan analysis was performed using the Kaplan-Meyer survival analysis. In the DCA diet group, sham flies were fed DCA (0.5 mg/mL) only for 1 week following needle injury and then switched back to regular diet. There was no lifespan difference between regular and DCA diet receiving sham flies (p > 0.05). (TIF) [file pone.0241122.s001.tif]
