# Supplementary material for: Dichloroacetate-induced metabolic reprogramming improves lifespan in a Drosophila model of surviving sepsis
Source: PLoS One. 2020 Nov 5;15(11):e0241122. doi: 10.1371/journal.pone.0241122 (PMC7643993; doi:10.1371/journal.pone.0241122)
Supplement: S1 Table — (DOCX) [file pone.0241122.s003.docx]

**S1 Table**

**RT-qPCR Settings**

| **Gene name** | **Gene symbol** | **UniGene ID** | **Flybase ID** | **TaqMan ID** | **Probe sequence (5’ to 3’)** | **Exon boundary** |
| --- | --- | --- | --- | --- | --- | --- |
| *toll* | Tl | Dm.2347 | FBgn0262473 | Dm02151201_g1 | GCATTAGCGGCGATATATTCAGCAA | 3-4 |
| *defensin* | Def | Dm.19893 | FBgn0010385 | Dm01818074_s1 | GGCTCAGCCAGTTTCCGATGTGGAT | 1-1 |
| *drosomycin* | Drs | Dm.14479 | FBgn0283461 | Dm01822006_s1 | CGCGTTTAGCTCTCCACTACTTACA | 1-1 |
| *cecropin A* | CecA1 | Dm.24116 | FBgn0000276 | Dm02609400_sH | CAATGTCGCCGCAACTGCCCGAGGT | 2-2 |
| *Actin 5C* | Act5C | Dm.2951 | FBgn0000042 | Dm02361909_s1 | GCGGAACCACCTGCACACCATCATC | 2-2 |

Probes and primers were ordered from Applied Biosystems as Assays-on-Demand (AoD)*****. AoD gene assays are provided with context sequence surrounding the assay location.

The PCR settings were

| **PCR Step** | **Setting** | | **Cycles** |
| --- | --- | --- | --- |
| *Polymerase activation and*  *DNA denaturation* | 95 °C for 30 s |  | 40 |
| *Amplification* |  | 95 °C for 5 s |  |
|  |  | 65 °C for 30 s |  |

***** The MIQE Guidelines: Minimum Information for Publication of Quantitative Real-Time PCR Experiments. Clin Chem2009 Apr;55(4):611-22. doi: [10.1373/clinchem.2008.112797](https://doi.org/10.1373/clinchem.2008.112797)
