# Supplementary material for: Dichloroacetate-induced metabolic reprogramming improves lifespan in a Drosophila model of surviving sepsis
Source: PLoS One. 2020 Nov 5;15(11):e0241122. doi: 10.1371/journal.pone.0241122 (PMC7643993; doi:10.1371/journal.pone.0241122)
Supplement: S2 Table — (DOCX) [file pone.0241122.s004.docx]

**S2 Table**

**LC-MS Settings**

| **Metabolite** | **CAS number** | **Molecular formula** | **Molecular weight** | ***m/z*** | **mode** |
| --- | --- | --- | --- | --- | --- |
| *Lactate* | 79-33-4 | C_3_H_6_O_3_ | 90.08 | 224.0671 | negative |
| *Pyruvate* | 113-24-6 | C_3_H_3_O_3_ | 88.06 | 357.0947 | negative |
| *Acetyl CoA* | 72-89-9 | C_23_H_38_N_7_O_17_P_3_S | 809.57 | 810.1330 | *positive* |
| *Citrate/Isocitrate* | 320-77-4 | C_6_H_8_O_7_ | 192.1235 | 596.1489 | negative |
| *α-Ketoglutarate* | 328-50-7 | C_5_H_6_O_5_ | 146.11 | 550.1434 | negative |
| *Acetate* | 71-50-1 | C_2_H_3_O_2_ | 59.04 | 194.0574 | negative |
| *Succinate* | 110-15-6 | C_4_H_6_O_4_ | 118.09 | 387.1053 | negative |
| *Fumarate* | 110-17-8 | C_4_H_4_O_4_ | 116.07 | 385.0896 | negative |
| *Malate* | 617-48-1 | C_4_H_6_O_5_ | 134.09 | 403.1002 | negative |

*m/z* range was 150 – 900 and the following

<https://pubmed.ncbi.nlm.nih.gov/23580203/>
